# Prostaglandin E_2_ stimulates opposing effects on inner and outer blood-retina barrier function

**DOI:** 10.3389/fphar.2025.1608376

**Published:** 2025-09-26

**Authors:** Amy K. Stark, Anna R. Gilmartin, Cayla D. Ontko, Taylor E. Smith, Amanda L. Beall, Gary W. McCollum, John S. Penn

**Affiliations:** ^1^ Department of Pharmacology, Vanderbilt University, Nashville, TN, United States; ^2^ Department of Ophthalmology and Visual Sciences, Vanderbilt University Medical Center, Nashville, TN, United States

**Keywords:** ophthalmology, diabetic retinopathy, diabetic macular edema, prostaglandin, lipid signaling, inflammation, blood-retina barrier, barrier function

## Abstract

**Introduction:**

Diabetic retinopathy (DR) is the leading cause of vision loss in working-age individuals globally, and the associated complication of diabetic macular edema (DME) is the most frequent cause of vision loss in these patients. The retinal swelling characteristic of DME can be attributed to fluid leakage due to damage to the two blood-retina barriers—the inner barrier composed primarily of retinal microvascular endothelial cells and the outer barrier composed of retinal pigment epithelial cells (RPE).

**Methods:**

Based on the previously characterized proinflammatory roles of prostanoid signaling in DR, we assayed the distinct prostanoid signaling mechanisms regulating inner and outer blood-retina barrier function using *in vitro* methods involving monoculture of primary human cells.

**Results:**

Prostaglandin E2 (PGE2) stimulation of retinal endothelial monolayers caused a decrease in barrier permeability in electric cell-substrate impedance sensing (ECIS) assays and dextran flux assays. These effects occurred via the EP4 receptor of PGE2. In direct contrast, PGE2 stimulation of RPE monolayers caused an increase in barrier permeability via the EP2 receptor. Other prostanoids did not alter barrier permeability in either monocellular model. RNA sequencing of retinal endothelial and RPE cells with or without PGE2 stimulation revealed significant dysregulation of genes encoding junctional complex components and signaling that likely drive the observed effects on cell barrier resistance.

**Discussion:**

Together these results suggest opposing mechanisms of PGE2 signaling in the retina via two distinct receptors, indicating cell type-specific and likely receptor-specific targets for the potential therapeutic management of DME or other causes of dysfunctional retinal vascular permeability.

## 1 Introduction

In the United States and worldwide, diabetic retinopathy (DR), a microvascular complication of diabetes mellitus, has become a leading cause of irreversible vision loss (Yau et al., 2012; Lundeen et al., 2023; Kempen et al., 2004). DR pathogenesis is characterized in its early stages by retinal features that include rising inflammation, pericyte and endothelial cell death, capillary regression, neuronal damage, leukocyte adhesion, and vascular leakage from blood-retina barrier dysfunction (Tang and Kern, 2011; Antonetti et al., 2012; Antonetti et al., 2021; Bianco et al., 2022). Together, these features define the stage of nonproliferative diabetic retinopathy (NPDR) (Tang and Kern, 2011; Antonetti et al., 2012). As the disease progresses, aberrant angiogenesis of retinal capillaries, known as neovascularization, marks the onset of proliferative diabetic retinopathy (PDR), the later stage of disease that is responsible for the most severe and irreversible vision loss (Tang and Kern, 2011; Antonetti et al., 2012).

Notably, swelling of the retina known as diabetic macular edema (DME) is the most common cause of vision loss in patients with diabetes, although in some cases it is reversible with treatment and can even resolve spontaneously (Ferris and Patz, 1984). DME is defined as the accumulation of fluid and lipids within the layers of the retina particularly in the macula, the central region responsible for high acuity vision (Ferris and Patz, 1984; Bhagat et al., 2009). Worsening conditions of diabetes and DR cause breakdown of the blood-retina barriers and imbalances in retinal fluid intake and drainage that lead to swelling and retinal thickening (Ferris and Patz, 1984; Bhagat et al., 2009; Daruich et al., 2018). In one study, up to 44% of diabetic patients developed some degree of DME when monitored over 9 years (Diabetes Control and Complications Trial Research Group, 1995). DME can develop in patients with any stage of retinopathy, but it is increasingly observed as DR severity and time since diabetes onset increases (Klein et al., 1984; Klein et al., 1995).

The role of inflammation in the early phases of DR and DME has emerged as a key area of research to study and treat disease. In particular, the prostanoid family of signaling lipids generated by cyclooxygenase (COX)-1 and COX-2-mediated metabolism of arachidonic acid may be especially relevant to the progression of retinal vascular diseases (Stark and Penn, 2024a). The five prostanoids signal through 9 G protein-coupled receptors (GPCRs) with different specificities and distinct downstream signaling: prostaglandins PGD_2_ via receptors DP1 and DP2; PGE_2_ via EP1, EP2, EP3, and EP4; PGF_2α_ via FP; PGI_2_ via IP; and thromboxane A_2_ (TXA_2_) via TP. Due to the many downstream pathways that could be activated by diverse prostanoid signaling, we hypothesize that select prostanoids and/or their receptors may be clinically relevant targets for the management of inflammation in DME.

Retinal vascular hyperpermeability, a key feature of DME, may affect both the inner and outer barriers of the retina. The inner blood-retina barrier is primarily composed of the retinal microvascular endothelial cells that form the vessels supplying blood to the inner retina. Tight junction proteins connect neighboring endothelial cells, and these cells function in coordination with retinal pericytes and Müller glia as the neurovascular unit of the retina (Antonetti et al., 2012; Pan et al., 2021; O'Leary and Campbell, 2023). The outer blood-retina barrier is composed of the retinal pigment epithelial cells (RPE) and serves as the physical barrier between the photoreceptors and the fenestrated capillary bed of the choroid. Adjacent RPE are also interconnected by tight junction proteins to regulate fluid and nutrient transport from the choroid and back (Pan et al., 2021; O'Leary and Campbell, 2023).

Work from Nakamura et al. previously studied prostanoid signaling in blood-retina barriers from a perspective of clinical utility (Nakamura et al., 2023). Latanoprost, the common glaucoma treatment that facilitates uveoscleral outflow and lowers intraocular pressure, is an analog of PGF_2α_. Similarly, the more recently approved glaucoma drug omidenepag isopropyl is a PGE_2_-EP2 receptor agonist. The retinal inflammatory effects of these two drugs were evaluated by Nakamura and colleagues in human retinal microvascular endothelial cells (hRMEC) and ARPE-19 cells to model the inner and outer blood-retina barriers, respectively. Co-stimulation with both latanoprost and omidenepag, but not either drug alone, increased proinflammatory cytokine levels in each cell type. Notably, co-stimulation also enhanced barrier function of hRMEC, yet conversely, it decreased barrier function of ARPE-19 (Nakamura et al., 2023).

Here, we sought to fully characterize the pharmacology of prostanoid signaling in the inner and outer blood-retina barriers using *in vitro* techniques. We observed changes in the cell barrier permeability of both hRMEC and RPE cultures treated with PGE_2_ but not when treated with PGF_2α_. Interestingly, the PGE_2_ effects were observed in opposite directions: PGE_2_ strengthened hRMEC barrier resistance via the EP4 receptor, but it reduced ARPE-19 resistance via the EP2 receptor, each occurring through elevated cAMP signaling. RNA sequencing of hRMEC or ARPE-19 with or without PGE_2_ stimulation revealed differential expression of many genes involved in junctional complexes, possible mechanisms for these changes in barrier resistance. Together, these new results suggest cell type-specific, receptor-specific responses to DR-relevant prostanoid signaling with potential implications for the management of DME.

## 2 Materials and methods

### 2.1 Materials and reagents

Catalog numbers and supplier information for all reagents are presented in Supplementary Table S1.

### 2.2 Cell culture

Primary human retinal microvascular endothelial cells (hRMEC) were obtained from Cell Systems. Cells were grown on tissue culture-treated dishes coated with Attachment Factor (Cell Systems) in Endothelial Basal Media (EBM; Cell Systems) supplemented with 10% fetal bovine serum (FBS; R&D Systems; Lot #M22319) and EGM SingleQuots (Lonza). Passage 8 cells were used for experiments.

Primary human retinal pigment epithelial cells (hRPE) were obtained from Lonza. Cells were grown on uncoated tissue culture-treated dishes in Dulbecco’s Modified Eagle Medium/Nutrient Mixture F-12 (DMEM/F-12; Gibco) supplemented with 10% FBS and 1% penicillin/streptomycin (Gibco). Passage 5 cells were used for experiments.

Human RPE cell line ARPE-19 cells were obtained from ATCC. Cells were grown in DMEM/F-12 supplemented with 10% FBS and 1% penicillin/streptomycin on uncoated tissue culture-treated dishes for all experiments except for ECIS assays, where wells were coated with Attachment Factor. Passage 24–25 cells were used for experiments.

All cell cultures were maintained in incubators held at 37 °C, 5% CO_2_, and 95% humidity and used FBS from a single lot, #M22319, to reduce uncontrolled experimental variability.

### 2.3 Electric cell-substrate impedance sensing (ECIS) assays

ECIS assays were performed in an ECIS Z-Theta 96-well array station (Applied BioPhysics). Gold electrode culture plates (Applied BioPhysics) were incubated with 10 mM L-cysteine (Sigma-Aldrich) for 10 min then washed twice with cell culture grade water (Sigma-Aldrich). Culture plates were then coated with Attachment Factor before plating cells. Assays were run with the ECIS array station housed in a cell culture incubator maintained at 37 °C, 5% CO_2_, and 95% humidity for the duration of the experiments. Media were changed daily until treatment. When cell monolayer resistance reached a stable plateau after 18–72 h, cells were treated with PGD_2_ (10 pM-10 μM), PGE_2_ (3 pM-10 μM), PGF_2α_ (3 pM-10 μM), DP1 antagonist BW A868C (50 nM–500 nM), DP2 antagonist OC000459 (50 nM–500 nM), EP1 antagonist SC-51322 (5 nM–500 nM), EP2 antagonist PF-04418948 (30 pM-3 μM), EP2 antagonist TG4-155 (100 nM-10 μM), EP3 antagonist DG-041 (5 nM–500 nM), EP4 antagonist L-161,982 (10 pM-10 μM), EP2 agonist butaprost (1 nM-10 μM), EP4 agonist L-902,688 (1 pM-100 nM), FP antagonist AL8810 (50 nM–500 nM), IP antagonist CAY10441 (50 nM–500 nM), TP antagonist daltroban (50 nM–500 nM), forskolin (1 μM), phosphodiesterase inhibitor IBMX (300 μM), PKA inhibitor KT5720 (1 μM), EPAC inhibitor ESI-09 (10 μM), or relevant vehicles. Treatments were diluted in DMSO so equal concentrations of DMSO were present in each sample per experiment, never exceeding 0.2% of the final volume. No cytotoxicity or negative effects were observed under 0.2% DMSO. In experiments using receptor antagonists or IBMX, cells were pretreated with the relevant inhibitor for 3 h followed by PGE_2_ stimulation. Following treatment, resistance of monolayers at 4,000 Hz of stimulation was analyzed. Resistance measures of each well were normalized to the resistance of that well immediately prior to treatment (time 0).

### 2.4 Transwell dextran flux assays

Transwell assays were performed using polyester membrane transwell insert plates, 0.4 μm pore size, 12 mm diameter or 6.5 mm diameter (Corning). For hRMEC assays, the transwell inserts were coated with Attachment Factor prior to plating. For ARPE-19 assays, phenol-free DMEM/F-12 (Gibco) supplemented as described above was used. Cells were grown to confluence in the top chamber with fresh media in the bottom chamber. Where applicable, cells were pretreated for 3 h by replacing media in the top and bottom chambers with media containing the EP4 antagonist L-161,982 (1 μM) or DMSO vehicle. For treatment, media in the bottom chambers were replaced with media containing the relevant treatment of (10 nM–100 nM PGE_2_ or DMSO vehicle ± antagonist), and media in the top chamber were replaced with relevant treatment +1 mg/mL FITC-conjugated 70 kDa dextran (Sigma-Aldrich). Fluorescence in the bottom chamber was measured with 492 nm excitation and 518 nM emission with a plate reader after 24 h of incubation. Cell-free transwell inserts in these conditions yielded 3.22-fold higher fluorescence values compared with vehicle-treated cell-coated transwell inserts (data not shown). Data were normalized to vehicle-treated conditions and reported as fold change in fluorescence vs vehicle.

### 2.5 RNA sequencing

hRMEC and ARPE-19 cultures were treated with 100 nM PGE_2_ or vehicle for 6 h (n = 3). After treatment, cells were washed with cold PBS, lysed, and total RNA was extracted using the RNeasy Mini Kit (Qiagen) according to the manufacturer’s protocol. RNA samples were submitted to the Vanderbilt Technologies for Advanced Genomics (VANTAGE) core laboratory (Nashville, TN) for RNA sequencing (RNA-Seq). Total RNA was quantified using a BioTek Synergy Multimode Plate reader and BioTek Gen5 2.09 software before being processed for RNA sequencing. RNA quality was determined using the Agilent Bioanalyzer 2,100 Instrument. RNA-Seq libraries were prepared using 200 ng of total RNA and the NEBNext rRNA Depletion Kit (New England Biolabs) according to manufacturer’s instructions. This kit employs an RNaseH-based method to deplete both cytoplasmic (5S rRNA, 5.8S rRNA, 18S rRNA, and 28S rRNA) and mitochondrial ribosomal RNA (12S rRNA and 16S rRNA) from total RNA preparations. The mRNA was enriched via poly-A-selection using oligoDT beads (New England Biolabs) and then the RNA was thermally fragmented and converted to cDNA. The cDNA was adenylated for adaptor ligation and PCR amplified. The libraries were sequenced using the Illumina NovaSeq 6,000 with 150 bp paired end reads targeting 50 M reads per sample. Illumina Real Time Analysis NovaSeq Control Software (1.8.0) was used for base calling. Gene transcripts were considered significantly differentially expressed if they met the inclusion criteria of a ±2 fold change and an adjusted *P*-value < 0.05.

### 2.6 Western blots

hRMEC and ARPE-19 cells were grown to confluence in 6-well tissue culture-treated plates (Corning). Cells were treated with 100 nM PGE_2_ or vehicle for 6 h, washed with cold PBS, and lysed in RIPA buffer (Sigma) containing cOmplete Mini EDTA-free Protease Inhibitor Cocktail tablets (Roche). Lysates were centrifuged at 10,000 x g for 10 min, then supernatants were isolated for analysis. The total protein concentrations were determined using a Pierce BCA assay kit. Equal concentrations of protein were loaded and run on 4%–20% Mini-PROTEAN TGX polyacrylamide gels (Bio-Rad) then transferred using the iBlot two system (Invitrogen) and nitrocellulose transfer stacks (Invitrogen). Blots were incubated in Intercept TBS blocking buffer (LI-COR) for 1 h and subsequently stained with the primary antibodies rat anti-Frizzled-4 (1 μg/mL), rabbit anti-ZO-2 (1 μg/mL), rabbit anti-CLDND1 (0.25 μg/mL), mouse anti-occludin (1 μg/mL), rabbit anti-PAR-3 (0.5 μg/mL), or mouse anti-β-actin (1:2000), each diluted in blocking buffer with 0.2% Tween 20 (Sigma) as specified. Four washes in TBS with 0.1% Tween 20 were performed, then blots were stained with the secondary antibodies 800CW donkey anti-rabbit (1:10,000), 680LT donkey anti-mouse (1:10,000), or 680RD goat anti-rat (1:10,000). Images were captured using a LI-COR Odyssey CLx reader and quantified using Fiji/ImageJ. Protein levels were normalized to β-actin and reported as fold-change versus vehicle-treated samples.

### 2.7 cAMP ELISA

ARPE-19 cells were grown to confluence in 96-well flat-bottom tissue culture-treated plates (Corning). Cells were pretreated for 1 h with the EP2 antagonist PF-04418948 (100 nM-1 μM) and then stimulated with PGE_2_ (1 μM) for 15 min. Samples were lysed and cAMP was quantified using a cAMP competitive ELISA (Abcam) according to the kit’s instructions.

### 2.8 Statistical analyses

Data analyses were performed using GraphPad Prism 10 software. Data are represented as mean ± standard deviation (SD) shown as error bars. For ECIS assays, where applicable, area under the curve (AUC) was calculated for the average resistance of each treatment group over 12 h. One-way ANOVAs with Tukey’s post hoc multiple comparison tests were used to compare AUC results with relevant comparisons shown. Dose-response curves were fit for relevant experiments using a three-parameter nonlinear regression, and the relative IC_50_ or EC_50_ values were calculated from these curves. For transwell dextran flux assays, data were analyzed using unpaired T-tests (for two groups) or one-way ANOVAs with Tukey’s or Dunnett’s post hoc multiple comparison tests (for three groups, as specified). For the cAMP ELISA, one outlier was removed by a Grubbs’ outlier identification with an alpha of 0.01, then data were analyzed by one-way ANOVA with Tukey’s post hoc multiple comparison tests. The threshold for significance for all assays was *P* < 0.05.

## 3 Results

### 3.1 PGE_2_ decreases permeability of hRMEC monolayers modeling the inner blood-retina barrier

**FIGURE 1:**
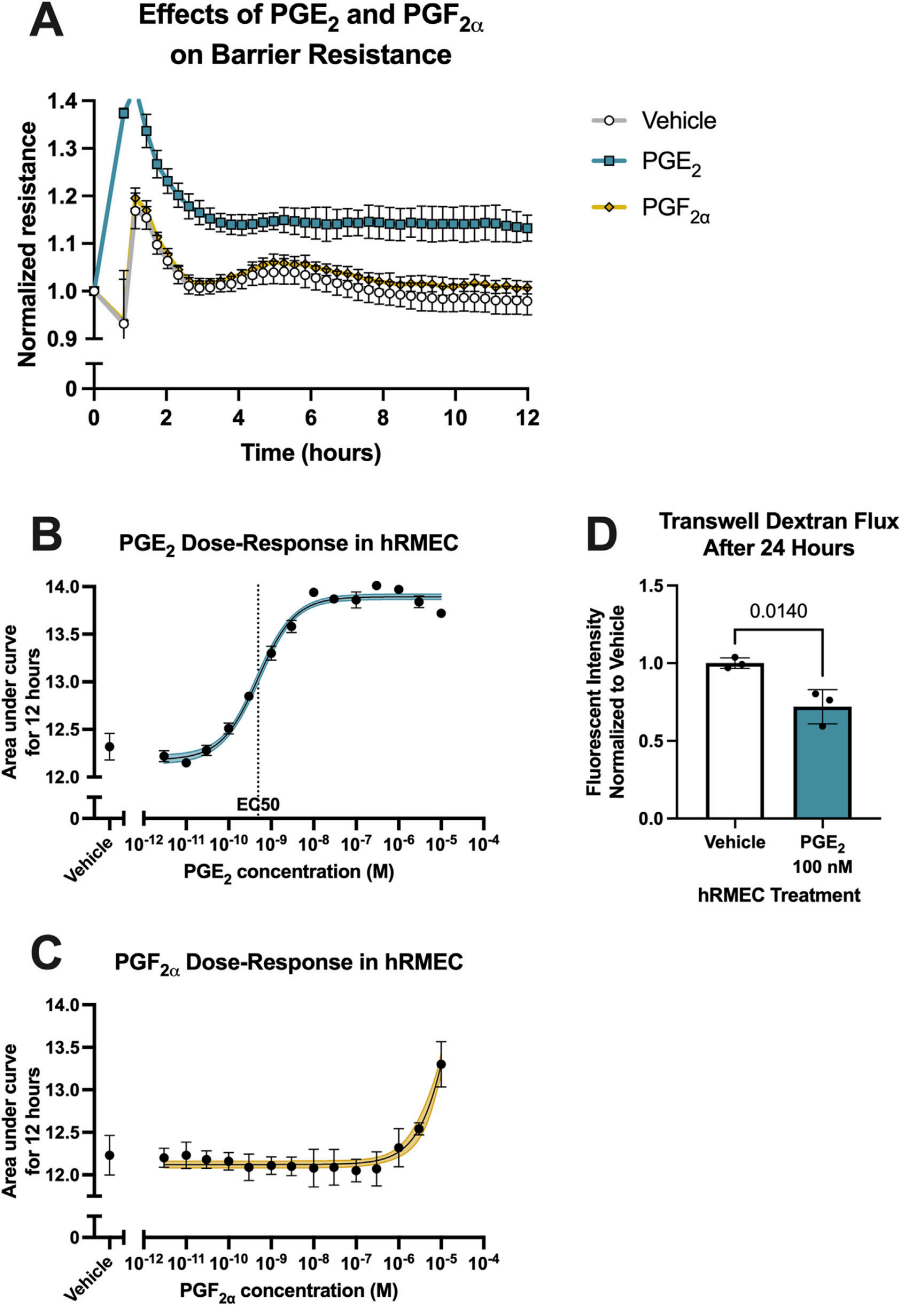
PGE_2_, but not PGF_2α_, strengthens hRMEC barrier function. **(A)** Normalized resistance ECIS results of hRMEC stimulated with 100 nM PGE_2_, 100 nM PGF_2α_, or vehicle over 12 h (n = 8–16, passage 8). **(B)** Dose-response curve of hRMEC stimulation with 3 pM–10 μM PGE_2_ or vehicle over 12 h (n = 6–12, passage 8). **(C)** Dose-response curve of hRMEC stimulation with 3 pM–10 μM PGF_2α_ or vehicle over 12 h (n = 6-8, passage 8). **(D)** Transwell dextran flux across hRMEC stimulated with 100 nM PGE_2_ or vehicle after 24 h (n = 3, passage 8). All data represent mean ± SD shown by error bars. 1B-C were analyzed using three-parameter nonlinear regression models with the 95% confidence intervals shown. 1D was analyzed using an unpaired T-test with the *P*-value shown.

To evaluate the effects of prostanoid stimulation on inner blood-retina barrier function, the resistance of hRMEC monolayers was measured with ECIS assays. Cells were grown in ECIS plates to a steady-state resistance indicative of mature monolayers before prostanoid or vehicle stimulation. Previous work from our laboratory found that primary human Müller glia, which aid in damage responses and maintaining homeostasis of the retina, cultured in conditions modeling systemic diabetes elevate production of PGE_2_, whereas hRMEC increased production of PGF_2α_ in these conditions (Stark and Penn, 2024b). Here, 100 nM PGF_2α_ promoted a very modest increase in hRMEC monolayer ECIS resistance over 12 h, whereas 100 nM PGE_2_ rapidly increased hRMEC monolayer resistance and sustained this effect for the full assay period (Figure 1A; Supplementary Figure S1A). These effects were further characterized across a wide range of prostanoid concentrations. Area under the curve (AUC) measurements from the dose-response of hRMEC stimulated with PGE_2_ for 12 h indicates an EC_50_ of 491.2 pM for PGE_2_ (Figure 1B). In contrast, the dose-response curve for PGF_2α_ was flat over the physiologic range, only showing a change in resistance with 3 μM or 10 μM, high concentrations that are unlikely to be specific to the FP receptor of PGF_2α_ (Figure 1C). As further confirmation of the effects of PGE_2_, transwell dextran flux assays were performed. 100 nM PGE_2_ reduced FITC-conjugated 70 kDa dextran flux across hRMEC monolayers by 27.94% after 24 h, validating the barrier enhancement observed in ECIS experiments (Figure 1D).

### 3.2 Inner blood-retina barrier enhancement is mediated by the EP4 receptor

**FIGURE 2:**
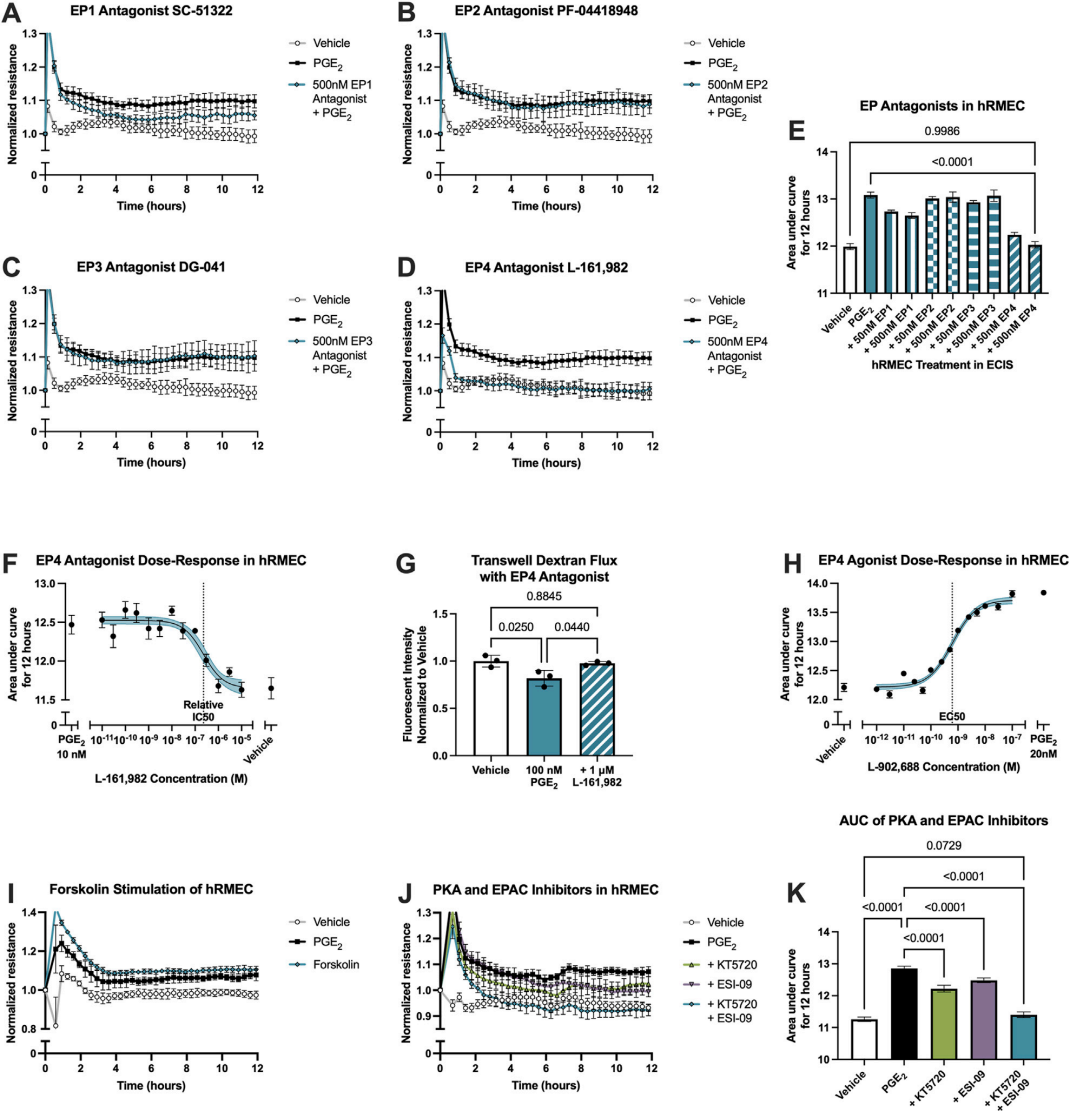
PGE_2_-induced barrier enhancement of hRMEC is mediated by the EP4 receptor. Normalized ECIS resistance measures from hRMEC pretreated with **(A)** 500 nM SC-51322, **(B)** 500 nM PF-04418948, **(C)** 500 nM DG-041, **(D)** 500 nM L-161,982, or vehicle for 3 h followed by stimulation with 10 nM PGE_2_ or vehicle over 12 h (n = 6-7, passage 8). Each antagonist is separated to an individual graph for clarity. **(E)** ECIS results expressed as AUC over 12 h from treatment of hRMEC with 50 nM–500 nM of EP antagonists +10 nM PGE_2_ or vehicle (n = 6-7, passage 8, comparisons to 500 nM EP4 antagonist shown). **(F)** Dose-response curve of hRMEC pretreated with 10 pM-10 μM L-161,982 or vehicle for 3 h followed by 10 nM PGE_2_ or vehicle stimulation over 12 h (n = 3-8, passage 8). **(G)** Transwell dextran flux across hRMEC pretreated with 1 μM L-161,982 or vehicle followed by 100 nM PGE_2_ or vehicle stimulation after 24 h (n = 3, passage 8). **(H)** Dose-response curve of hRMEC stimulation with 1 pM–100 nM L-902,688, 20 nM PGE_2_, or vehicle over 12 h (n = 4-6, passage 8). **(I)** Normalized resistance ECIS results of hRMEC stimulated with 1 μM forskolin, 10 nM PGE_2_, or vehicle over 12 h (n = 5-6, passage 8). **(J)** Normalized resistance ECIS results of hRMEC pretreated with 1 μM KT5720, 10 μM ESI-09, 1 μM KT5720 + 10 μM ESI-09, or vehicle for 3 h followed by 10 nM PGE_2_ or vehicle stimulation over 12 h (n = 5-6, passage 8). **(K)** ECIS results expressed as AUC over 12 h from treatment of hRMEC ± KT5720 and ESI-09 + 10 nM PGE_2_ or vehicle (n = 5-6, passage 8). All data represent mean ± SD shown by error bars. 2E, 2G, and 2K were analyzed using one-way ANOVAs and Tukey’s multiple comparisons tests with *P*-values shown for relevant comparisons. 2F and 2H were analyzed using three-parameter nonlinear regression models with the 95% confidence intervals shown.

PGE_2_ signals via four GPCRs with distinct downstream signaling. Antagonists selective for each receptor were employed to determine through which receptor(s) PGE_2_ promotes the enhancement of hRMEC barrier function. Antagonists tested were SC-51322 for EP1, PF-04418948 for EP2, DG-041 for EP3, and L-161,982 for EP4. hRMEC were pretreated with vehicle or an antagonist for 3 h followed by stimulation with 10 nM PGE_2_. The EP1 receptor antagonist SC-51322 at 500 nM had modest effects in inhibiting PGE_2_-induced elevation of ECIS resistance in hRMEC, but the EP4 receptor antagonist L-161,982 at 500 nM completely inhibited these effects of PGE_2_ on barrier function (Figures 2A–E). Lower concentrations of each antagonist were tested but not shown in 2A-D due to lesser or lack of efficacy. The EP4 antagonism was confirmed within a range of L-161,982 concentrations, and an IC_50_ of 229.6 nM was determined from the dose-response curve (Figure 2F). In complementary transwell dextran flux assays, pretreatment of 1 μM L-161,982 for 1 h fully inhibited the decrease in permeability observed by stimulation with 100 nM PGE_2_ (Figure 2G). Additionally, the EP4 agonist L-902,688 promoted barrier enhancement in ECIS with an EC_50_ of 613.5 pM, mimicking the full effects of PGE_2_ in hRMEC (Figure 2H).

Downstream signaling of the EP4 receptor, a Gα_s_-coupled receptor that promotes adenylyl cyclase (AC) activation and cAMP production, was examined by comparing the effects of PGE_2_ stimulation to the effects of the direct AC activator forskolin in an ECIS assay. 1 μM forskolin promoted a comparable elevation of barrier resistance to that caused by 10 nM PGE_2_ stimulation, suggesting that the PGE_2_-induced change in resistance is cAMP-dependent (Figure 2I; Supplementary Figure S1B). Finally, further signaling activation downstream of cAMP was evaluated using the protein kinase A (PKA) inhibitor KT5720 (1 μM), the EPAC inhibitor ESI-09 (10 μM), or both inhibitors in combination in ECIS assays. hRMEC were pretreated with inhibitor(s) or vehicle for 3 h followed by 10 nM PGE_2_ stimulation. Both the PKA and EPAC inhibitors partially yet significantly reduced PGE_2_-induced barrier resistance elevation. The combination of both inhibitors prevented this change in resistance to levels not significantly different from vehicle-treated wells by AUC measurement, indicating a full blockade of downstream signaling (Figures 2J,K). Therefore, EPAC and PKA are dual downstream effectors of PGE_2_-EP4-cAMP barrier enhancement in hRMEC.

### 3.3 PGE_2_ dysregulates junctional complexes in hRMEC

**FIGURE 3:**
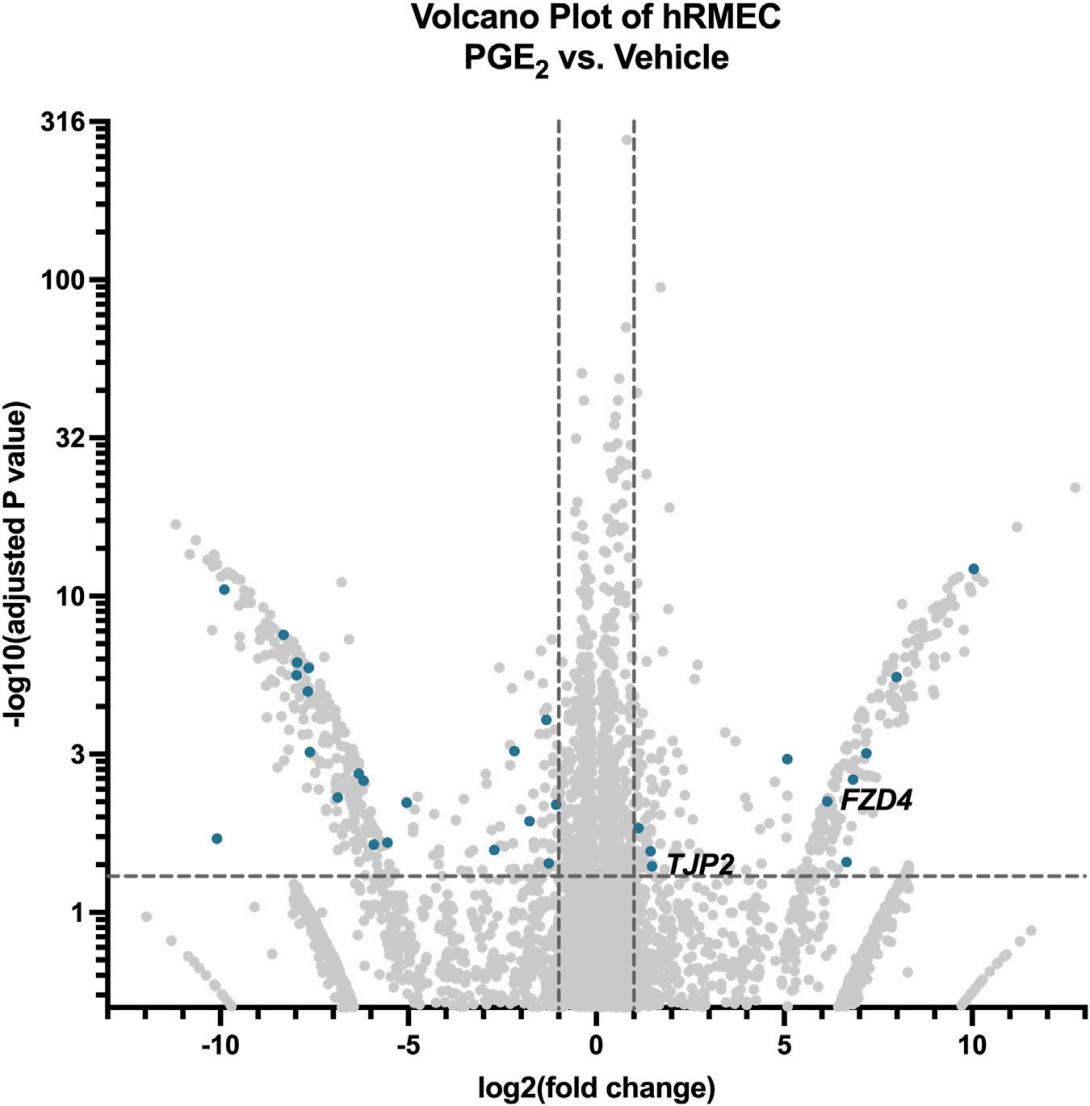
RNA-seq of hRMEC reveals differential gene expression after PGE_2_ stimulation. Volcano plot of differentially expressed genes and transcripts in hRMEC after 6-h stimulation with 100 nM PGE_2_ compared with vehicle controls. Blue dots represent differentially expressed transcripts involved in tight junctions and/or cell-cell junctions. Labels to the right of dots highlight hypothesized important gene expression changes. Transcripts upregulated or downregulated by 2-fold with an adjusted *P*-value < 0.05 were considered significantly differentially expressed.

To explore the mechanism by which PGE_2_ strengthens hRMEC barrier function, we performed bulk RNA sequencing of hRMEC stimulated with 100 nM PGE_2_ or vehicle for 6 h. There were 616 differentially expressed gene transcripts that met the inclusion criteria of a fold change greater than two or less than −2 and an adjusted *P*-value less than 0.05. From these data, we identified 30 transcripts annotated with the Gene Ontology terms “tight junction” or “cell-cell junction” that were differentially expressed after PGE_2_ treatment (Figure 3). Transcripts specifically involved in tight junctions, as filtered with the Gene Ontology term “tight junction,” are shown in Table 1. Most relevant from this dataset is the 2.80-fold elevation of *TJP2* (ZO-2), as well as the 70.62-fold elevation of *FRZ4* (Frizzled-4). Upregulation of these known tight junction-related components as well as other genes could explain the alteration in hRMEC barrier resistance driven by PGE_2_. Western blots to validate these targets at the protein level showed a nonsignificant 39.6% increase in Frizzled-4 levels after PGE_2_ stimulation but no change in ZO-2 levels, indicating additional junctional complex proteins and molecular mechanisms could also be involved in these effects Supplementary Figure S2A,S2B).

**TABLE 1:** Differentially expressed tight junction gene transcripts in PGE_2_-stimulated hRMEC.

Gene name	Transcript ID	log2FoldChange	Adjusted *P*-value
*FZD4*	ENST00000531380.2	6.1424	0.00570
*TJP2*	ENST00000650460.1	1.4877	0.03971
*TBCD*	ENST00000682921.1	−1.7747	0.01149
*CDK4*	ENST00000552388.1	−2.7092	0.02656
*C1QTNF5*	ENST00000528368.3	−5.5547	0.02182
*CCND1*	ENST00000227507.3	−6.1937	0.00249
*ECT2*	ENST00000441497.6	−6.8792	0.00492
*SYNPO*	ENST00000307662.5	−7.9699	2.4E-06

### 3.4 PGE_2_ increases permeability of RPE monolayers modeling the outer blood-retina barrier

**FIGURE 4:**
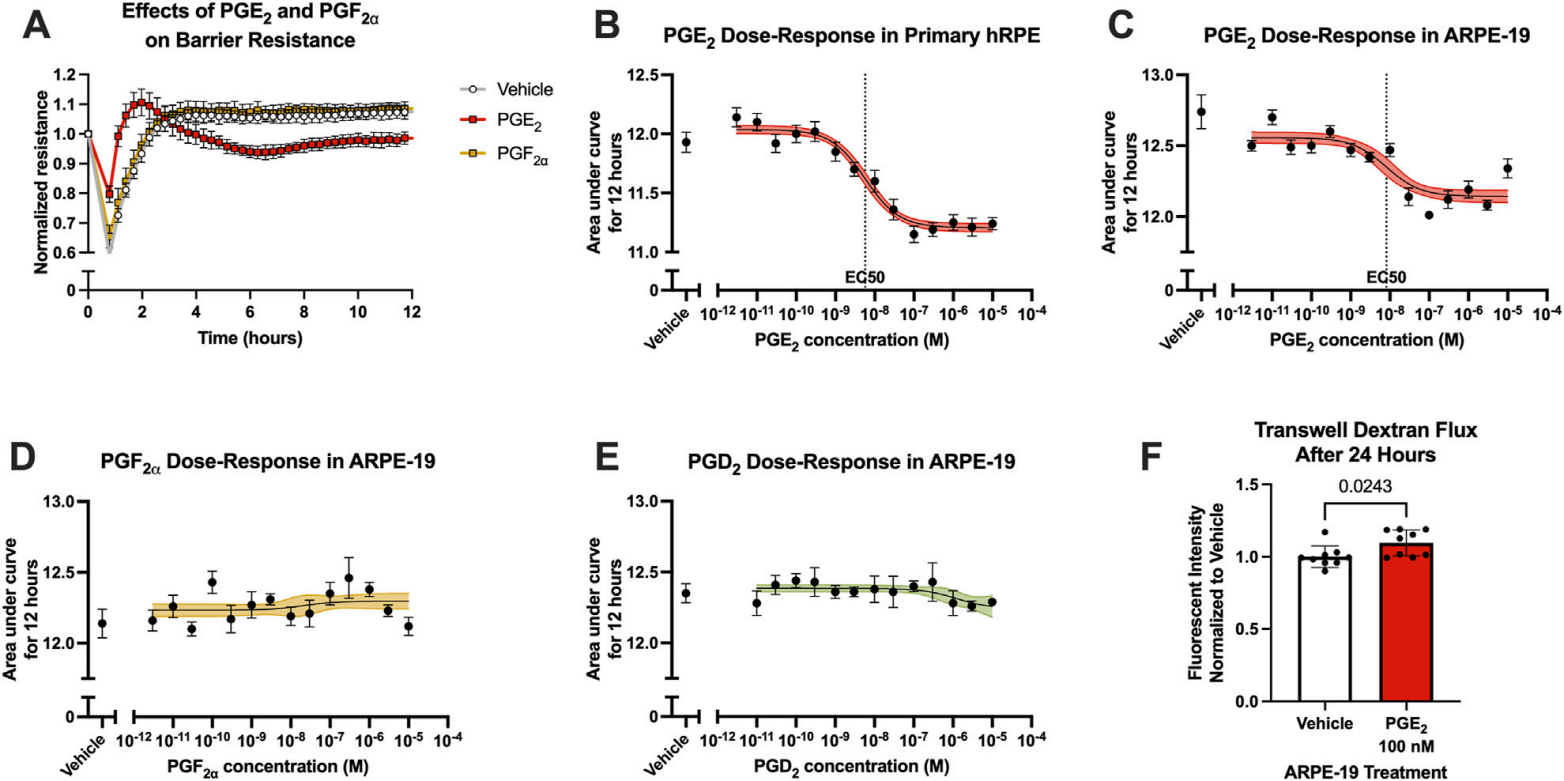
PGE_2_, but not PGF_2α_, induces RPE barrier permeability. **(A)** Normalized resistance ECIS results of hRPE stimulated with 100 nM PGE_2_, 100 nM PGF_2α_, or vehicle over 12 h (n = 6, passage 5). **(B)** Dose-response curve of hRPE stimulation with 3 pM–10 μM PGE_2_ or vehicle over 12 h (n = 6-9, passage 5). **(C)** Dose-response curve of ARPE-19 stimulation with 3 pM–10 μM PGE_2_ or vehicle over 12 h (n = 5–11, passage 24). **(D)** Dose-response curve of ARPE-19 stimulation with 3 pM–10 μM PGF_2α_ or vehicle over 12 h (n = 6-9, passage 24). **(E)** Dose-response curve of ARPE-19 stimulation with 3 pM–10 μM PGD_2_ or vehicle over 12 h (n = 4-6, passage 24). **(F)** Transwell dextran flux across ARPE-19 stimulated with 100 nM PGE_2_ or vehicle after 24 h (n = 9, passage 24). All data represent mean ± SD shown by error bars. 4B-E were analyzed using three-parameter nonlinear regression models with the 95% confidence intervals shown. 4F was analyzed using an unpaired T-test with the *P*-value shown.

Because of the complex differences between the inner and outer blood-retina barriers, the effects of prostanoid signaling on outer blood-retina barrier function was also investigated using RPE cell culture models in ECIS assays. Primary human RPE cells (hRPE) were cultured in ECIS plates to a steady-state resistance characteristic of mature monolayers, then cells were stimulated with prostanoids or vehicle. Similar to hRMEC, hRPE resistance was largely unaffected by 100 nM PGF_2α_ stimulation. However, 100 nM PGE_2_ had an opposite effect in hRPE to that observed in hRMEC: it promoted a transient decrease in ECIS resistance, indicating an increase in barrier permeability, that recovered to vehicle levels after 24 h (Figure 4A; Supplementary Figure S1C).

Stimulation of hRPE with a range of PGE_2_ concentrations yielded a complete dose-response curve with a calculated EC_50_ of 5.672 nM as measured using AUC of ECIS results over 12 h (Figure 4B). However, hRPE have significant limitations resulting from their responses to culture conditions, affecting barrier forming properties, altering cell morphology, and causing rapid epithelial-mesenchymal transition at low passages. Therefore, we continued these assays using the well-characterized immortalized human RPE cell line, ARPE-19 cells. The transcriptional and functional properties of ARPE-19 also differ from hRPE, notably including a higher proliferative capacity of ARPE-19 that could facilitate robust monolayer formation essential for ECIS assays (Alge et al., 2006). The parallel dose-response assay in ARPE-19 generated an EC_50_ of 8.135 nM for PGE_2_, indicating comparable effects on barrier function in the two RPE cell culture models despite a partially reduced threshold for change in resistance (Figure 4C). The resistance of ARPE-19 stimulated with PGE_2_ began to return to baseline over time whereas resistance of hRPE stimulated with PGE_2_ remained decreased for the duration of the experiments, likely due to the different proliferative capacities of these cell types. Stimulation with either PGF_2α_ or PGD_2_ up to 10 μM showed no change on ARPE-19 barrier resistance, suggesting PGE_2_ selectivity of this permeability effect (Figures 4D,E). Furthermore, stimulation of ARPE-19 cells with 100 nM PGE_2_ in transwell dextran flux assays caused a 9.6% increase in permeability after 24 h, similar to the maximal induction of permeability in ECIS (Figure 4F).

### 3.5 Outer blood-retina barrier permeability is mediated by the EP2 receptor

**FIGURE 5:**
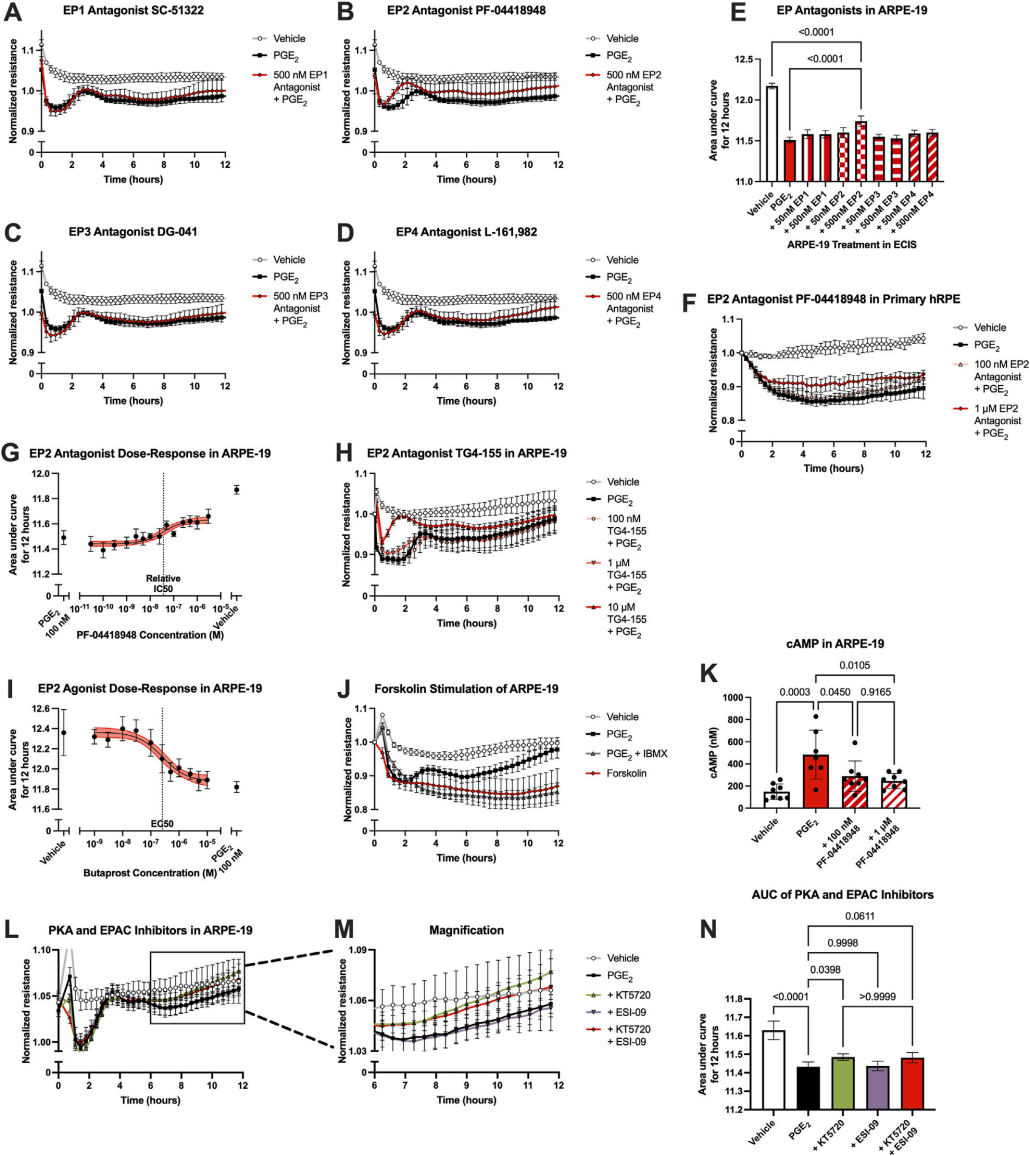
PGE_2_-induced barrier permeability of ARPE-19 is mediated in part by the EP2 receptor. Normalized resistance ECIS results of ARPE-19 pretreated with **(A)** 500 nM SC-51322, **(B)** 500 nM PF-04418948, **(C)** 500 nM DG-041, **(D)** 500 nM L-161,982, or vehicle for 3 h followed by stimulation with 100 nM PGE_2_ or vehicle over 12 h (n = 4-6, passage 25). Each antagonist is separated to an individual graph for clarity. **(E)** ECIS results expressed as AUC over 12 h from treatment of ARPE-19 with 50 nM–500 nM of EP antagonists +100 nM PGE_2_ or vehicle (n = 4-6, passage 25, comparisons to 500 nM EP2 antagonist shown). **(F)** Normalized resistance ECIS results of hRPE pretreated with 100 nM-1 μM PF-04418948 for 3 h followed by stimulation with 100 nM PGE_2_ or vehicle over 12 h (n = 4-6, passage 5). **(G)** Dose-response curve of ARPE-19 pretreated with 30 pM-3 μM PF-04418948 or vehicle for 3 h followed by 100 nM PGE_2_ or vehicle stimulation over 12 h (n = 3-6, passage 24). **(H)** Normalized resistance ECIS results of ARPE-19 pretreated with 100 nM-10 μM TG4-155 or vehicle for 3 h followed by stimulation with 100 nM PGE_2_ over 12 h (n = 5-8, passage 25). **(I)** Dose-response curve of ARPE-19 stimulation with 1 nM–10 μM butaprost, 100 nM PGE_2_, or vehicle over 12 h (n = 6–10, passage 24). **(J)** Normalized resistance ECIS results of hRMEC pretreated with 300 μM IBMX or vehicle for 3 h followed by stimulation with 1 μM forskolin, 100 nM PGE_2_, or vehicle over 12 h (n = 5-6, passage 24). **(K)** cAMP production in ARPE-19 pretreated with 100 nM-1 μM PF-04418948 for 1 h followed by stimulation with 1 μM PGE_2_ or vehicle for 15 min (n = 7-8, passage 24). **(L)** Normalized resistance ECIS results of ARPE-19 pretreated with 1 μM KT5720, 10 μM ESI-09, 1 μM KT5720 + 10 μM ESI-09, or vehicle for 3 h followed by 100 nM PGE_2_ or vehicle stimulation over 12 h (n = 6, passage 24). **(M)** Magnification of KT5720 ± ESI-09 ECIS results from 6 to 12 h. **(N)** ECIS results expressed as AUC over 12 h from treatment of hRMEC ± KT5720 and ESI-09 + 10 nM PGE_2_ or vehicle (n = 6, passage 24). All data represent mean ± SD shown by error bars. 5E, 5K, and 5N were analyzed using one-way ANOVAs and Tukey’s multiple comparisons tests with *P*-values shown for relevant comparisons. 5G and 5I were analyzed using three-parameter nonlinear regression models with the 95% confidence intervals shown.

The receptor(s) mediating the effects of PGE_2_ on ARPE-19 barrier permeability were probed using selective antagonists, as analyzed in hRMEC. Here, ARPE-19 were pretreated with vehicle or an antagonist for 3 h, then cells were stimulated with 100 nM PGE_2_. In these assays, only the EP2 receptor antagonist PF-04418948 at 500 nM inhibited PGE_2_-induced permeability (Figures 5A–E). Lower antagonist concentrations were tested but not shown in 5A-D due to lack of efficacy. As confirmation, PGE_2_-induced permeability in primary hRPE was also inhibited to a similar degree by PF-04418948 (Figure 5F; Supplementary Figure S1D). However, dose-response curves generated from a range of PF-04418948 concentrations in ARPE-19 revealed that the maximal effects of this antagonist partially prevented the effects of PGE_2_, up to 44.7% recovery with a relative IC_50_ of 36.7 nM (Figure 5G). These partial effects were also confirmed using a second EP2-selective antagonist, TG4-155 (Figure 5H; Supplementary Figure S1E). Stimulation with the EP2 selective agonist butaprost did model the compete effects of PGE_2_ to promote barrier permeability with an EC_50_ of 256.4 nM (Figure 5I). Therefore, while PGE_2_ may also signal via an unidentified receptor in RPE, EP2 is the primary receptor mediating the increase in resistance.

As EP2, like EP4, is a Gα_s_-coupled receptor, the effects of PGE_2_ were also compared to forskolin stimulation to determine if these effects were mimicked by cAMP elevation. Indeed, 1 μM forskolin induced permeability to a similar extent as 100 nM PGE_2_ with a 3-h pretreatment of 300 μM IBMX, a phosphodiesterase inhibitor to prevent cAMP degradation, suggesting cAMP dependence in this cell behavior (Figure 5J; Supplementary Figure S1F). Furthermore, ELISAs for cAMP levels showed that 1 μM PGE_2_ increased cAMP production 226% over vehicle treatment, and PF-04418948 partially but significantly prevented PGE_2_-stimulated cAMP production to a similar degree as observed in previous experiments: 40.3% reduction for 100 nM and 49.3% reduction for 1 μM PF-04418948 versus PGE_2_ (Figure 5K). Lastly, signaling downstream of cAMP was also evaluated using PKA inhibitor KT5720, EPAC inhibitor ESI-09, or their combination in ECIS assays. In ARPE-19, only KT5720 inhibited the PGE_2_-induced decrease in resistance to a degree, restoring resistance to baseline by 12 h post-stimulation and showing a small yet significant effect by AUC measurement. ESI-09 had no effect in ARPE-19, and the combination of inhibitors was not different from KT5720 alone (Figures 5L–N). Together, these results indicate that PGE_2_ induces barrier permeability in RPE via EP2-cAMP-PKA signaling.

**FIGURE 6:**
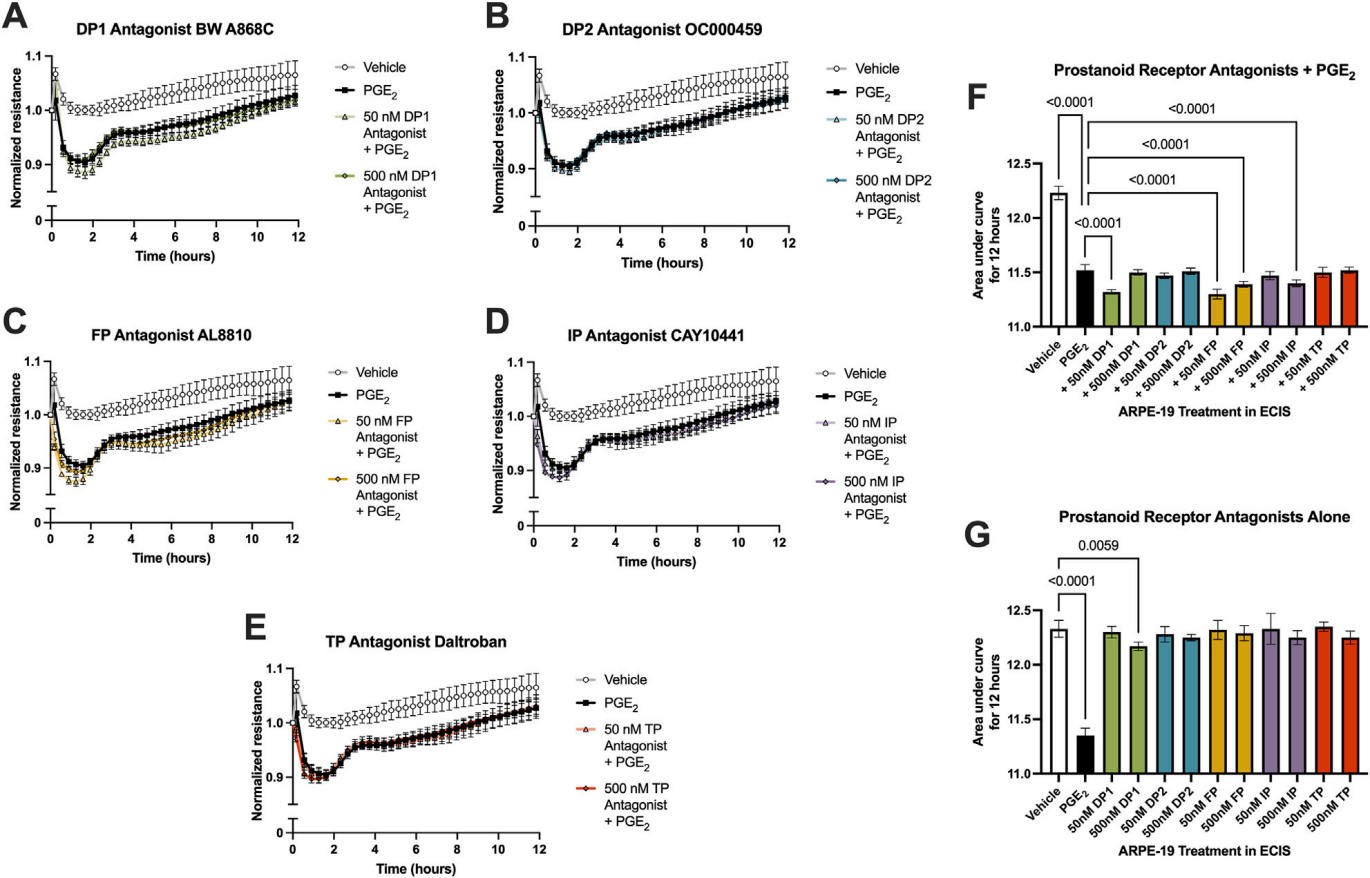
PGE_2_ does not signal off-target via a non-EP prostanoid receptor in ARPE-19. Normalized ECIS resistance measures from ARPE-19 pretreated with **(A)** 50–500 nM BW A868C, **(B)** 50–500 nM OC000459, **(C)** 50–500 nM AL8810, **(D)** 50–500 nM CAY10441, **(E)** 50–500 nM daltroban, or vehicle for 3 h followed by stimulation with 100 nM PGE_2_ or vehicle over 12 h (n = 4-6, passage 24). Each antagonist is separated to an individual graph for clarity. **(F)** ECIS results expressed as AUC over 12 h from treatment of ARPE-19 with 50 nM–500 nM of EP antagonists +100 nM PGE_2_ or vehicle (n = 4-6, passage 24). **(G)** ECIS results expressed as AUC over 12 h from treatment of ARPE-19 with 100 nM PGE_2_, vehicle, or 50 nM–500 nM of EP antagonists alone (n = 5-6, passage 24). All data represent mean ± SD shown by error bars. 6F was analyzed using a one-way ANOVA with Dunnett’s multiple comparisons test to compare all groups to 100 nM PGE_2_ treatment, significant *P*-values shown. 6G was analyzed using a one-way ANOVA with Dunnett’s multiple comparisons test to compare all groups to vehicle treatment, significant *P*-values shown.

Because the permeability-inducing effects of PGE_2_ were only partially inhibited by EP2 receptor antagonists yet antagonists to EP1, EP3, and EP4 did not affect this signaling, off-target signaling of PGE_2_ via other prostanoid receptors was evaluated in ARPE-19 ECIS assays. Stable ARPE-19 monolayers were pretreated for 3 h with the antagonists BW A868C for the DP1 receptor, OC000459 for the DP2 receptor, AL8810 for the FP receptor, CAY10441 for the IP receptor, and daltroban for the TP receptor as well as relevant vehicles, followed by stimulation with 100 nM PGE_2_ or vehicle. None of these other prostanoid receptor antagonists up to 500 nM concentrations prevented PGE_2_-induced permeability (Figures 6A–F). Of note, 50 nM DP1, 50 nM FP, 500 nM FP, and 500 nM IP antagonists did cause small but statistically significant decreases in the AUC measures over 12 h compared with PGE_2_ alone (Figure 6F). This indicates minor exacerbation of PGE_2_-induced permeability. These may represent small but genuine physiological effects of these antagonists. Nevertheless, none of these antagonists inhibit the effects of PGE_2_ on ARPE-19 permeability, thus suggesting that PGE_2_ does not induce permeability by off-target signaling via another non-EP prostanoid receptor. Furthermore, treatment with antagonists alone (no PGE_2_ stimulation) did not elicit significantly different AUC values from vehicle-treated cells with the exception of 500 nM DP1, which again yielded only a very small induction of permeability (Figure 6G, individual traces not shown). We conclude that EP2 is the primary prostanoid receptor of action for PGE_2_-induced permeability in RPE because only antagonists to the EP2 receptor demonstrated a mitigating effect on PGE_2_-induced monolayer resistance.

### 3.6 PGE_2_ dysregulates junctional complexes in ARPE-19

**FIGURE 7:**
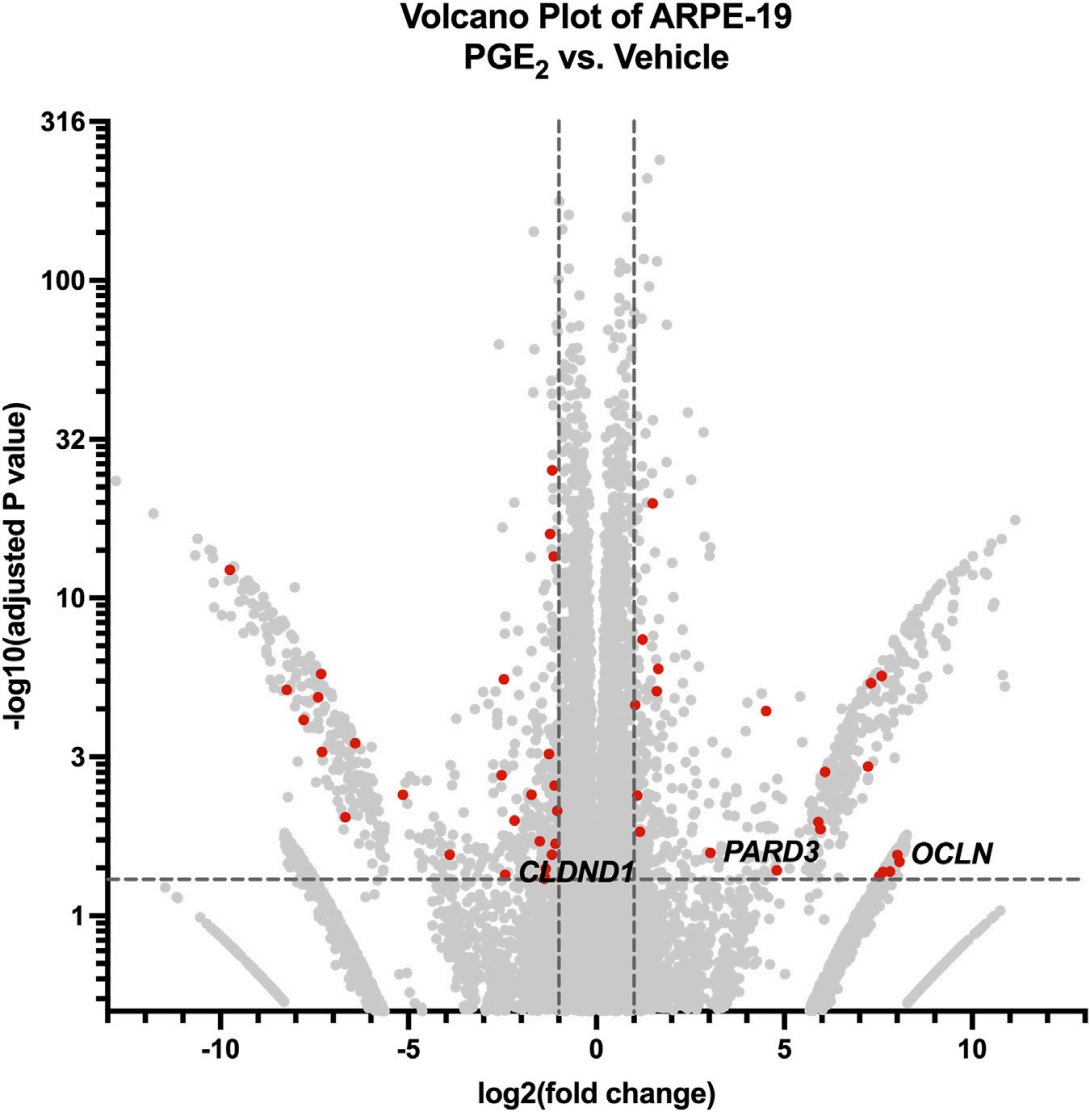
RNA-seq of ARPE-19 reveals differential gene expression after PGE_2_ stimulation. Volcano plot of differentially expressed genes and transcripts in ARPE-19 after 6-h stimulation with 100 nM PGE_2_ compared with vehicle controls. Red dots represent differentially expressed transcripts involved in tight junctions and/or cell-cell junctions. Labels to the right of dots highlight hypothesized important gene expression changes. Transcripts upregulated or downregulated by 2-fold with an adjusted *P*-value < 0.05 were considered significantly differentially expressed.

Finally, we conducted bulk RNA sequencing of ARPE-19 samples stimulated with 100 nM PGE_2_ or vehicle for 6 h to study mechanisms of PGE_2_-induced barrier permeability in these cells. Here, 1,354 differentially regulated genes and transcripts met the aforementioned inclusion criteria, and 48 of these transcripts were annotated with the Gene Ontology “tight junction” or “cell-cell junction” terms (Figure 7). The 15 transcripts representing nine unique genes filtered with the “tight junction” Gene Ontology term are shown in Table 2. Out of these differentially expressed genes, the 257.9-fold upregulation of *OCLN* (occludin), 8.19-fold upregulation of *PARD3* (PAR-3), and 5.39-fold downregulation of *CLDND1* (claudin domain-containing protein 1) may well cause the defects in ARPE-19 barrier function observed after PGE_2_ stimulation. Western blots of ARPE-19 validated significant occludin upregulation and CLDND1 downregulation after PGE_2_ stimulation, yet PAR-3 protein levels were unchanged (Supplementary Figures S2C,S2D). Therefore, additional molecular mechanisms are also likely involved in this blood-retina barrier regulation.

**TABLE 2:** Differentially expressed tight junction gene transcripts in PGE_2_-stimulated ARPE-19.

Gene name	Transcript ID	log2FoldChange	Adjusted *P*-value
*ASH1L*	ENST00000368346.7	8.0636	0.0332
*OCLN*	ENST00000514370.5	8.0106	0.0279
DLG1*	ENST00000667104.1	7.6326	0.0422
*UBN1*	ENST00000585857.1	6.0854	0.0015
DLG1*	ENST00000419227.5	5.8992	0.0106
*RPGRIP1L*	ENST00000262135.9	4.5196	3.88E-05
*PARD3*	ENST00000696673.1	3.0345	0.0264
DLG1*	ENST00000448528.6	−1.2308	1.19E-16
DLG1*	ENST00000419354.5	−1.2642	0.0006
DLG1*	ENST00000664564.1	−1.3700	0.0474
DLG1*	ENST00000661013.1	−1.3784	0.0492
*ARHGEF2*	ENST00000462460.6	−1.5016	0.0192
DLG1*	ENST00000665728.1	−1.7221	0.0039
*CLDND1*	ENST00000506927.1	−2.4310	0.0450
*PMP22*	ENST00000395938.7	−9.7495	5.52E-13

* denotes gene with multiple differentially expressed transcripts.

## 4 Discussion

In this study, we analyzed the effects of prostanoid stimulation of cells that regulate retinal barrier function as a potential component of DME disease onset and progression. DME progression is most widely studied from the perspective of inner blood-retina barrier leakage, yet the outer blood-retina barrier function could also play an important role in this condition. One study analyzing FITC-dextran leakage from both the inner and outer blood-retina barriers in streptozotocin (STZ)-induced diabetic mice showed a relative ratio of leakage of 1:2.48 from the outer versus inner barriers, showing that a significant portion of dextran leakage occurred via the outer blood-retina barrier (Xu and Le, 2011). This high degree of vascular leakage attributable to each barrier warranted further study of these barriers together in the context of DR and DME.

Previous work from our laboratory indicated that two prostanoids, PGE_2_ and PGF_2α_, are produced significantly and consistently by primary human Müller glia and retinal microvascular endothelial cells, respectively, when cultured in conditions modeling the chronic hyperglycemia, dyslipidemia, and inflammation prevalent systemically in diabetes (Stark and Penn, 2024b). This informed our selection of stimuli for investigation relevant to retinal barrier function here.

PGF_2α_ has pronounced roles in the anterior chamber of the eye, yet its role in DME is less characterized. The PGF_2α_ analogs latanoprost, bimatoprost, travoprost, and tafluprost, formulated as topical eyedrops used once daily, serve as the standard-of-care therapy for glaucoma (Aihara, 2021). These drugs act by increasing uveoscleral outflow of aqueous humor from the anterior segment of the eye to decrease intraocular pressure (Toris et al., 1993). Despite recognition of its roles in glaucoma, PGF_2α_ in DR pathologies is less studied and understood. Our previous work showed that PGF_2α_-FP signaling can stimulate proinflammatory cytokine production in Müller glia as well as leukocyte adhesion to hRMEC monolayers, each important pathologies in early DR (Stark and Penn, 2024b). Additionally, PGF_2α_ signaling has well recognized roles in pericyte death, another critical event occurring early in DR. Bovine retinal pericytes cultured in conditions of elevated glucose showed impaired adhesion to culture matrices and migration through transwell chambers, and stimulation with PGF_2α_ restored both functions via FP receptor/RhoA signaling (Peng et al., 2018). Further, intraperitoneal latanoprost injection prevented STZ-induced capillary regression in the superficial—but not deep—capillary plexus of the mouse retina, indicating functional consequences of FP-mediated pericyte survival (Peng et al., 2018). These investigators subsequently found that human retinal pericytes demonstrated elevated apoptosis when cultured in elevated glucose conditions, and PGF_2α_ also prevented glucose-induced cell death via the FP receptor’s activation of the PI3K/Akt/GSK3β/β-catenin pathway (Cheng et al., 2021). These other effects relevant to early-stage DR notwithstanding, there are no prior reports of involvement of PGF_2α_ signaling in either inner or outer blood-retina barrier function *in vitro*. This suggests that effects of prostanoid signaling in the retina are cell type-specific and behavior-specific.

In contrast, many effects of PGE_2_ in cell behaviors of DR have been characterized. Relevant to NPDR, PGE_2_ stimulated *in vivo* vascular leakage, retinal thickening, endothelial cell apoptosis, capillary dropout, and leukocyte adhesion in STZ rats (Wang et al., 2019). The EP2 agonist butaprost mimicked these effects, and AH6809 inhibited them (Wang et al., 2019); however, AH6809 is a prostaglandin antagonist with comparable affinity to the EP1, EP2, EP3, and DP1 receptors (Abramovitz et al., 2000), and the specificity of its effects to one receptor were not evaluated in that study. In our previous work, we showed that PGE_2_-EP2 signaling promoted proinflammatory cytokine production in primary human Müller glia but not leukocyte adhesion to hRMEC (Stark and Penn, 2024b). PGE_2_ also has noted proangiogenic roles relevant to PDR via both EP3 (Sennlaub et al., 2003; Chen et al., 2017) and EP4 (Yanni et al., 2009; Xie et al., 2021) receptor signaling. Furthermore, PGE_2_ levels in vitreous samples from patients with PDR were 53% higher than the levels from nondiabetic patient vitreous samples (Schoenberger et al., 2012). In this study using well-characterized antagonists, each with selectivity for a single EP receptor, we determined that PGE_2_ strengthens barrier function in hRMEC via the EP4 receptor and decreases barrier function in ARPE-19 via the EP2 receptor, results that are partially aligned with these previous studies.

The effects of PGE_2_ on barrier function have been documented in other systems with similarly distinct effects in endothelial or epithelial cell types. Directly compared with our findings in hRMEC, lung microvascular endothelial cells have similarly strengthened barrier function via PGE_2_-EP4 signaling in ECIS and other transendothelial resistance assays (Birukova et al., 2007; Konya et al., 2013; Bärnthaler et al., 2017). Still, not all endothelial cell types respond in this manner. PGE_2_ caused an increase in dextran flux across human brain microvessel endothelial cell monolayers in transwell chambers, an effect that was attenuated by either EP3 or EP4 antagonists (Dalvi et al., 2015). In modeling the colon epithelium with T84 human colonic cells, PGE_2_ stimulated a decrease in transepithelial resistance via the EP4 receptor, a similar effect to our RPE results albeit via a different Gα_s_-coupled EP receptor (Lejeune et al., 2010). Further, signaling of PGE_2_ through both EP1 and EP4 promoted barrier dysfunction in Caco-2 cells, which also model the colon epithelium (Martín-Venegas et al., 2006; Rodríguez-Lagunas et al., 2010). Nonetheless, research comparing the RPE barrier to other epithelial barriers of the body, such as the colon epithelium, is limited.

Our findings support a role of EP4 in mediating the full barrier-enhancing effects of PGE_2_ in hRMEC, yet EP2 antagonists only partially prevented PGE_2_-induced permeability in ARPE-19. No other EP receptor antagonists affected monolayer resistance when tested at concentrations up to 500 nM. Additionally, neither PGD_2_ nor PGF_2α_ affected RPE barrier resistance in physiological ranges, and antagonists to DP1, DP2, FP, IP, and TP receptors did not inhibit PGE_2_-induced permeability. While certain concentrations of some of these antagonists caused a small but significant exacerbation of permeability, these effects show no blockade of PGE_2_-induced permeability. These statistically significant effects of 50 nM DP1, 50 nM FP, 500 nM FP, or 500 nM IP receptor antagonists could be attributable to two primary reasons. First, they may be due to the structural similarities among prostanoids and their receptor binding affinities, activating signaling that promotes permeability similarly to PGE_2_ (Narumiya et al., 1999). Second, the antagonists may inhibit the endogenous PGD_2_, PGF_2α_, or PGI_2_ signaling in these cultures that otherwise maintain normal barrier function, thereby exacerbating the effects of PGE_2_ to a degree. Previous studies have shown that DP1 activation reduces permeability of mouse vascular endothelium in Evans blue measurements and of bovine aortic endothelial cells in FITC-dextran flux assays (Murata et al., 2008), and IP activation decreases permeability of intestine epithelium biopsies from patients with inflammatory bowel diseases (Pochard et al., 2021). Additionally, while AL8810 is commonly used as an antagonist to block FP receptor signaling and is the best available compound for this purpose, AL8810 is, in fact, a very weak partial agonist of the FP receptor with maximal activating efficacy of 19%–23% (Griffin et al., 1999). AL8810 has been shown to promote EGFR transactivation and MAPK/ERK1/2 signaling via biased mechanisms distinct from those of PGF_2α_, which may also explain the permeability effects we observed (Goupil et al., 2012). To understand the additional effect of PGE_2_ that is not mitigated by EP2 antagonism, we must consider additional non-prostanoid receptors. To date, PGE_2_ has not been described to bind to any non-prostanoid receptors; however, other prostanoid ligands bind non-prostanoid receptors under some conditions. One possibility is that PGE_2_ may act as a PPARγ agonist. Other studies show that the EP4 agonist L-902,688 (Li, 2018), PGI_2_ analogs (Falcetti et al., 2007), PGD_2_ (Yu et al., 1995), and the PGD_2_ metabolite 15d-PGJ_2_ (Yu et al., 1995; Kliewer et al., 1995) all activate PPARγ; however, we do not believe that identifying novel signaling mechanisms of PGE_2_ falls within the scope of this work. Overall, although EP2 antagonists did not fully block this response, we hypothesize that EP2 is the primary prostanoid receptor mediating this signaling in RPE.

Although the opposite effects of PGE_2_ on hRMEC and RPE barrier function occur via different receptors, downstream signaling pathways of both EP2 and EP4 promote elevation of cAMP levels. These opposing cell behaviors in response to elevated cAMP levels have been observed previously. Enhancement of multiple types of microvascular endothelial barriers by cAMP signaling is well-characterized (Adamso et al., 1998; He et al., 2000). Adenylyl cyclase activation at the plasma membrane, elevating the membrane-localized cAMP concentration, drives this endothelial barrier strengthening (Sayner et al., 2006). Specific to retinal microvascular endothelial cells, several studies have shown that barrier function is preserved and/or restored by cAMP activation of both PKA and EPAC-Rap1 pathways independently, similar to our findings (Birukova et al., 2007; van der Wijk et al., 2017; Ramos et al., 2018; Liu et al., 2019; Steinle, 2020; Birukova et al., 2008; Lorenowicz et al., 2008). The barrier-altering effects of cAMP in RPE are less characterized, but published results are consistent with its ability to decrease permeability in cell culture and animal models (Pavan et al., 2014; Wittchen and Hartnett, 2011; Wittchen et al., 2013). Other studies showed cAMP-mediated permeability in RPE occurs via EPAC-Rap1 signaling (Wittchen and Hartnett, 2011; Wittchen et al., 2013), but we found that only PKA, not EPAC, mediated these effects in ARPE-19 after PGE_2_ stimulation. While specific mechanisms remain to be characterized in full, we hypothesize that cell type-specific and prostanoid-stimulated differences in expression levels of adenylyl cyclases, phosphotidesterases, or A-kinase anchoring proteins (AKAPs) could each affect the local cAMP levels. This would promote distinct gradients of cAMP that in turn differentially activate PKA and/or EPAC, altering the cellular responses as we observed herein.

Using RNA sequencing of hRMEC and ARPE-19 stimulated in the presence or absence of PGE_2_, we identified widespread up- and downregulation of genes involved in cell-cell junctions, particularly tight junctions, to identify gene products that might promote changes in barrier resistance in each cell type. 100 nM concentrations of PGE_2_ were chosen to stimulate both hRMEC and ARPE-19 rather than the respective EC_50_ values based on the PGE_2_ dose-response curves for each cell type. This higher concentration showed the maximal effects in each cell type without excessive doses that may likely promote off-target effects, so we therefore reasoned it would show optimal effects in RNA sequencing analyses. While the 100 nM PGE_2_ concentration may be limit the overall physiologic relevance, both EP4 inhibition in hRMEC and EP2 inhibition in ARPE-19 were still fully or mostly efficacious against this high concentration in other assays shown. In hRMEC, *TJP2* and *FRZ4* upregulation in RNA-seq each may support the observed increase in barrier resistance. *TJP2* encodes ZO-2, a critical component of blood-retina barriers that is especially important for maintaining membrane rigidity (Naylor et al., 2019; Campbell and Humphries, 2013; Pinto-Duenas et al., 2024). Furthermore, the frizzled class receptor 4 encoded by *FRZ4* is the primary receptor of norrin, and the norrin signaling pathway is known to promote blood-retina barrier function *in vitro* and *in vivo* (Diaz-Coranguez et al., 2020; Zhang et al., 2023). It is also a receptor for the Wnt ligand, and Wnt/β-catenin signaling also has important roles in maintaining the inner blood-retina barrier (Yemanyi et al., 2021). Antibody-based pharmacologic activation of FZD4 was sufficient to restore blood-retina barrier function that was compromised in mouse models (Ding et al., 2023); therefore, it is possible that *FRZ4* gene overexpression may have similar enhancing effects on barrier function. In ARPE-19, downregulation of *CLDND1* as well as upregulation of *OCLN* and *PARD3* were identified by our sequencing analysis. Claudin family proteins are well-characterized components of tight junctions that regulate barrier integrity of RPE and other epithelial cells and also interact with additional junctional complex proteins including occludin (Findley and Koval, 2009; Rizzolo et al., 2011). While occludin is similarly an essential component of junctional complexes to promote blood-retina barrier regulation (O'Leary and Campbell, 2023; Campbell and Humphries, 2013; Goncalves et al., 2021; Murakami et al., 2009), such high upregulation as observed here could detrimentally disrupt normal junctional complex organization. Similarly, complex dysfunction may be promoted by the upregulation of PAR-3, which has been shown to critically regulate RPE barriers through its interactions with PAR-6 and PKCζ or with junctional adhesion mole (JAM) (Omri et al., 2013; Ebnet et al., 2001). It is possible that overexpression of such junctional complex signaling proteins is counterproductive to blood-retina barrier integrity. Indeed, lower expression of tight junction constituents does not necessarily result in a weaker barrier, nor does higher expression necessarily promote a stronger barrier. Either up- or downregulation could disrupt organization and downstream signaling, leading to impaired function of the junctional complex. Western blot analyses suggested that Frizzled-4 was elevated in hRMEC and validated that occludin and CLDND1 were dysregulated in ARPE-19 in agreement with the RNA-seq results, but additional molecules not assayed here are likely involved in the PGE_2_-induced changes in barrier function in either cell type. Subsequent experiments to further validate additional targets and to test the individual roles of these up- and downregulated tight junction molecules are warranted for a full understanding of the mechanisms of PGE_2_ signaling on barrier function in both hRMEC and ARPE-19.

Our discovery that PGE_2_ causes opposing effects on barrier resistance in hRMEC versus ARPE-19 aligns with the results of Nakamura et al. (2023), yet our respective findings regarding which prostanoid(s) are responsible for these behaviors differ somewhat. While Nakamura et al. found that only co-stimulation with latanoprost and omidenepag elicited changes in barrier permeability, we instead detail that PGE_2_ alone, not PGF_2α_, drives the effects with high potency. The evaluation of latanoprost and omidenepag in barrier function assays both alone and in combination informs important drug safety and clinical relevance, yet we hypothesize that the effects on barrier function elicited by Nakamura’s combination approach may be due to the higher cumulative concentration of the drugs as they activate EP receptors. This would suggest off-target signaling for latanoprost, mirroring the effects of very high PGF_2α_ concentrations in our assays.

The use of ECIS assays allows powerful, sensitive, and high-throughput measurements of changes in permeability in both retinal cell types *in vitro*. Further, the relevance of these experiments is improved through validation with physical barrier leakage analyses in transwell dextran flux assays. Nonetheless, *in vitro* studies in cell culture models are inherently limited. Cell growth requires artificial conditions that do not replicate retinal architecture and involve non-native growth supplementation. FBS supplementation was required to facilitate cell growth and survival in culture, yet FBS itself contains growth factors, proteins, hormones, and fatty acids including arachidonic acid that could affect experiment endpoints (Subbiahanadar Chelladurai et al., 2021; Lee et al., 2023). To control for this, we used a consistent supplementation of all media with 10% FBS and used FBS of the same lot number for all experiments. Further, our *in vitro* study is limited in its relevance to the intact diabetic retina. Previous studies have begun to analyze effects of prostanoid signaling using *in vivo* methods. Nakamura et al. observed that RPE flat mounts from mice intravitreally injected with omidenepag, latanoprost, or both drugs in combination had disrupted ZO-1 junctional complex staining compared with vehicle-injected mice, indicating impaired barrier function due to EP2 and/or FP receptor signaling (Nakamura et al., 2023). Similarly, Wang et al. found that PGE_2_ and the EP2 agonist butaprost each exacerbated Evans blue dye vascular leakage in retinal whole mounts from rats with STZ-induced diabetes, and the partially selective EP2 antagonist AH6809 prevented this leakage (Wang et al., 2019). Finally, a study by Amrite et al. indirectly addressed a role of prostanoid signaling in vascular leakage *in vivo* by injecting COX-2-selective celecoxib-containing microparticles into the eyes of STZ rats. The celecoxib microparticles caused a 40% decrease in retinal PGE_2_ levels as well as a 50% decrease in FITC-dextran leakage from the retinas compared with control microparticle injections, which potentially supports the findings of PGE_2_-induced permeability (Amrite et al., 2006). Each of these studies, however, only analyzed vascular leakage across a single blood-retina barrier. To better characterize potentially distinct roles of PGE_2_ in blood-retina barrier function as we observed *in vitro*, FITC-dextran measurements in retinal transverse sections, a method established by Xu and Le. (2011), could be performed alongside the relevant PGE_2_ antagonist injections, facilitating the direct comparison of effects on inner and outer blood-retina barriers *in vivo*.

Overall, we found that hRMEC modeling the inner blood-retina barrier and RPE cell cultures modeling the outer blood-retina barrier respond to PGE_2_ in opposing manners. hRMEC barrier function is strengthened by PGE_2_ signaling via the EP4 receptor, whereas ARPE-19 barriers become more permeable from PGE_2_ signaling via the EP2 receptor. Both receptors promote intracellular cAMP production, which may affect different downstream signaling pathways and/or junctional complex proteins in these two cell types. Our findings might impact the management of DR and DME due to the opposing cellular responses to PGE_2_, which is elevated in patients with DR. Cell type-specific and receptor-specific therapeutic development could balance the pro-strengthening, anti-permeability effects of PGE_2_ in the blood-retina barriers relevant to vascular leakage and DME progression.

## Data Availability

The datasets presented in this study can be found in online repositories. The names of the repository/repositories and accession number(s) can be found below: https://www.ncbi.nlm.nih.gov/, GSE301373.
