# Supplementary material for: Prostaglandin E2 stimulates opposing effects on inner and outer blood-retina barrier function
Source: Front Pharmacol. 2025 Sep 26;16:1608376. doi: 10.3389/fphar.2025.1608376 (PMC12511020; doi:10.3389/fphar.2025.1608376)
Supplement: Supplementary file 1 [file DataSheet1.docx]

Supplementary Material

# Supplementary Figure 1

**Supplementary Figure 1.** Area under the curve (AUC) measurements of ECIS results over 12 hours. All data represent mean +/- standard deviation shown as error bars. One-way ANOVAs with Tukey’s post-hoc multiple comparison tests with *P*-values for relevant comparisons are shown.

# Supplementary Figure 2

**Supplementary Figure 2.** Western blot quantification and representative blots of Frizzled-4 and ZO-2 in hRMEC and CLDND1, occludin, and PAR-3 in ARPE-19 normalized to β-actin, n = 6 each. All data represent mean +/- standard deviation shown as error bars. Data were analyzed using unpaired T-tests with the *P*-values shown.

# Supplementary Table 1: Materials

| **Item** | **Catalog Number** | **Vendor** | **Vendor Location** |
| --- | --- | --- | --- |
| Primary human retinal microvascular endothelial cells | ACBRI 181 | Cell Systems | Kirkland, Washington, USA |
| Attachment Factor | 4Z0-201 | Cell Systems | Kirkland, Washington, USA |
| Endothelial basal media | 4Z3-500-R | Cell Systems | Kirkland, Washington, USA |
| Fetal bovine serum | S11550 | R&D Systems | Minneapolis, Minnesota, USA |
| EGM SingleQuots | CC-4133 | Lonza | Basel, Switzerland |
| Primary human retinal pigment epithelial cells | 00194987 | Lonza | Basel, Switzerland |
| DMEM/F-12 | 11320-033 | Gibco | Grand Island, New York, USA |
| Penicillin/streptomycin | 15140-122 | Gibco | Grand Island, New York, USA |
| ARPE-19 cells | CRL2302 | ATCC | Manassas, Virginia, USA |
| ECIS 96-well plate | 96W10idf | Applied BioPhysics | Troy, New York, USA |
| L-cysteine | 168149 | Sigma-Aldrich | St. Louis, Missouri, USA |
| Cell culture grade water | 3500 | Sigma-Aldrich | St. Louis, Missouri, USA |
| PGD_2_ | 12010 | Cayman Chemical | Ann Arbor, Michigan, USA |
| PGE_2_ | 14010 | Cayman Chemical | Ann Arbor, Michigan, USA |
| PGF_2α_ | 16010 | Cayman Chemical | Ann Arbor, Michigan, USA |
| BW A868C | 12060 | Cayman Chemical | Ann Arbor, Michigan, USA |
| OC000459 | 12027 | Cayman Chemical | Ann Arbor, Michigan, USA |
| SC-51322 | 10010744 | Cayman Chemical | Ann Arbor, Michigan, USA |
| PF-04418948 | 15016 | Cayman Chemical | Ann Arbor, Michigan, USA |
| TG4-155 | 17639 | Cayman Chemical | Ann Arbor, Michigan, USA |
| DG-041 | 6240 | Tocris | Bristol, United Kingdom |
| L-161,982 | 10011565 | Cayman Chemical | Ann Arbor, Michigan, USA |
| Butaprost | 13740 | Cayman Chemical | Ann Arbor, Michigan, USA |
| L-902,688 | 10007712 | Cayman Chemical | Ann Arbor, Michigan, USA |
| AL8810 | 16735 | Cayman Chemical | Ann Arbor, Michigan, USA |
| CAY10441 | 1005186 | Cayman Chemical | Ann Arbor, Michigan, USA |
| Daltroban | 14061 | Cayman Chemical | Ann Arbor, Michigan, USA |
| Forskolin | 11018 | Cayman Chemical | Ann Arbor, Michigan, USA |
| IBMX | 13347 | Cayman Chemical | Ann Arbor, Michigan, USA |
| KT5720 | 10011011 | Cayman Chemical | Ann Arbor, Michigan, USA |
| ESI-09 | 19130 | Cayman Chemical | Ann Arbor, Michigan, USA |
| 12 mm diameter transwell insert plates | 3460 | Corning | Kennebunk, Maine, USA |
| 6.5 mm diameter transwell insert plates | 3470 | Corning | Kennebunk, Maine, USA |
| Phenol-free DMEM/F-12 | 21041-025 | Gibco | Grand Island, New York, USA |
| FITC-conjugated 70 kDa dextran | 46945 | Sigma-Aldrich | St. Louis, Missouri, USA |
| RNeasy Mini Kit | 74106 | Qiagen | Germantown, Maryland, USA |
| NEBNext rRNA Depletion Kit | E6310X | New England Biolabs | Ipswich, Massachusetts, USA |
| 6-well culture plates | 3516 | Corning | Kennebunk, Maine, USA |
| RIPA buffer | R0278 | Sigma-Aldrich | St. Louis, Missouri, USA |
| cOmplete Mini, EDTA-free Protease Inhibitor Cocktail | 04693159001 | Roche | Basel, Switzerland |
| Pierce BCA Protein Assay Kit | 23225 | Thermo Scientific | Waltham, Massachusetts, USA |
| 4-20% Mini-PROTEAN TGX polyacrylamide gels | 4561094 | Bio-Rad | Hercules, California, USA |
| iBlot 2 | IB21001 | Invitrogen | Waltham, Massachusetts, USA |
| iBlot 2 nitrocellulose transfer stacks, regular | IB23001 | Invitrogen | Waltham, Massachusetts, USA |
| Intercept TBS blocking buffer | 927-60001 | LI-COR | Lincoln, Nebraska, USA |
| Rat anti-Frizzled-4 | MAB194 | R&D Systems | Minneapolis, Minnesota, USA |
| Rabbit anti-ZO-2 | 71-1400 | Invitrogen | Waltham, Massachusetts, USA |
| Rabbit anti-CLDND1 | PA5-56273 | Invitrogen | Waltham, Massachusetts, USA |
| Mouse anti-occludin | 33-1500 | Invitrogen | Waltham, Massachusetts, USA |
| Rabbit anti-PAR-3 | PA5-56475 | Invitrogen | Waltham, Massachusetts, USA |
| Mouse anti-β-actin | 3700 | Cell Signaling | Danvers, Massachusetts, USA |
| IRDye 800CW donkey anti-rabbit IgG | 925-32213 | LI-COR | Lincoln, Nebraska, USA |
| IRDye 680LT donkey anti-mouse IgG | 925-68022 | LI-COR | Lincoln, Nebraska, USA |
| IRDye 680RD goat anti-rat IgG | 926-68076 | LI-COR | Lincoln, Nebraska, USA |
| Tween 20 | P1379 | Sigma-Aldrich | St. Louis, Missouri, USA |
| 96-well culture plates | 3596 | Corning | Kennebunk, Maine, USA |
| cAMP competitive ELISA | ab234585 | Abcam | Waltham, Massachusetts, USA |
